# OGG1 Inhibitor TH5487 Alters OGG1 Chromatin Dynamics and Prevents Incisions

**DOI:** 10.3390/biom10111483

**Published:** 2020-10-26

**Authors:** Bishoy M. F. Hanna, Thomas Helleday, Oliver Mortusewicz

**Affiliations:** 1 Science for Life Laboratory, Department of Oncology-Pathology, Karolinska Institutet, 171 77 Stockholm, Sweden; bishoy.hanna@ki.se (B.M.F.H.); thomas.helleday@ki.se (T.H.); 2 Weston Park Cancer Centre, Department of Oncology and Metabolism, University of Sheffield, Sheffield S10 2RX, UK

**Keywords:** DNA oxidative damage, base excision repair, TH5487, OGG1 glycosylase inhibitor, chromatin dynamics, recruitment kinetics, 8-oxoguanine incision, γH2AX

## Abstract

8-oxoguanine DNA glycosylase (OGG1) is the main DNA glycosylase responsible for the excision of 7,8-dihydro-8-oxoguanine (8-oxoG) from duplex DNA to initiate base excision repair. This glycosylase activity is relevant in many pathological conditions including cancer, inflammation, and neurodegenerative diseases. To have a better understanding of the role of OGG1, we previously reported TH5487, a potent active site inhibitor of OGG1. Here, we further investigate the consequences of inhibiting OGG1 with TH5487. TH5487 treatment induces accumulation of genomic 8-oxoG lesions. Furthermore, it impairs the chromatin binding of OGG1 and results in lower recruitment of OGG1 to regions of DNA damage. Inhibiting OGG1 with TH5487 interferes with OGG1′s incision activity, resulting in fewer DNA double-strand breaks in cells exposed to oxidative stress. This study validates TH5487 as a potent OGG1 inhibitor that prevents the repair of 8-oxoG and alters OGG1–chromatin dynamics and OGG1′s recruitment kinetics.

## 1. Introduction

Reactive oxygen species (ROS) can be generated from a number of endogenous or exogenous sources. Endogenously generated ROS are a by-product of normal oxygen metabolism in different organelles including mitochondria, peroxisomes, and the endoplasmic reticulum. Exposure to ionizing radiation or some chemicals are among the exogenous sources of ROS [1]. Multiple lines of evidence indicate that loss of balanced redox homeostasis is implicated in many pathological conditions, e.g. cancer, neurodegenerative diseases, cardiovascular diseases, ischemia reperfusion injury, and inflammatory diseases, in addition to aging [2,3,4,5,6]. When maintained at appropriate cellular concentrations, ROS are involved in several physiological processes such as signaling pathways and immune responses [3,7]. However, at higher concentrations, ROS can overwhelm the cellular antioxidant capacity, generating oxidative stress and causing oxidative damage to lipids, proteins and DNA [1,3,4]. 

Owing to its low redox potential, guanine is the most vulnerable nucleobase to oxidation [8,9,10]. The major product of guanine oxidation is 7,8-dihydro-8-oxoguanine (8-oxoG), a mutagenic lesion that can mispair to adenine, leading to base transversions and potential genetic mutations [11]. 8-oxoguanine DNA glycosylase (OGG1) is the main glycosylase catalyzing 8-oxoG excision from duplex DNA when it is paired to cytosine to initiate base excision repair (BER) [12,13]. Recently, we reported the development of TH5487, a potent small-molecule active-site OGG1 inhibitor and provided proof of concept that targeting oxidative DNA repair by inhibiting OGG1 might serve as a beneficial strategy to alleviate inflammation [14]. 

Here, we aimed to further characterize TH5487 in terms of its capacity to inhibit DNA repair. In addition, we evaluated the inhibitor’s effect on OGG1–chromatin dynamics and how it impacts DNA incision. We observed that TH5487 treatment results in the accumulation of genomic 8-oxoG lesions. Furthermore, TH5487 reduces OGG1–chromatin binding and results in lower recruitment of OGG1 to regions of DNA damage. Inhibiting OGG1 with TH5487 impairs OGG1′s incision activity, resulting in fewer DNA double-strand breaks in oxidatively stressed cells. Taken together, our results indicate that TH5487 inhibits OGG1, preventing the repair of 8-oxoG, and alters OGG1–chromatin dynamics and OGG1′s recruitment kinetics.

## 2. Materials and Methods 

### 2.1. Cell Culture and Treatments

U2OS cells were cultured at 37 °C in a 5% CO_2_ atmosphere in Dulbecco’s Modified Eagle Medium (DMEM; Gibco) supplemented with 10% of fetal bovine serum (FBS; Gibco) and 100 U/mL penicillin–streptomycin (Gibco). To induce oxidative DNA damage, cells at about 80% confluency were treated with potassium bromate (Sigma-Aldrich CAS 7758-01-2) in serum-free DMEM for the indicated period of time. To inhibit OGG1, 10 μM TH5487 or 0.1% dimethyl sulfoxide (DMSO; VWR Chemicals) was used according to the indicated experimental scheme for the appropriate time periods. 

### 2.2. Generation of Stable Cell Lines Expressing OGG1-GFP 

U2OS cells were transfected with the hOGG1-GFP vector described in Visnes et al. [14] using jetPEI (Polyplus) and selected with 1 μg/mL puromycin for 10 days. To minimize variability in the expression levels, clonal expansion was carried out to generate a single clone of U2OS cells constitutively expressing GFP-tagged OGG1.

### 2.3. 8-oxoG Immunofluorescence Assay

Cells were seeded on an Ibidi 6-channel µ-Slide VI 0.4 (Ibidi, Catalog No. 80606) 24 h prior to the indicated treatment. Next, 20 mM potassium bromate (KBrO_3_; Sigma-Aldrich CAS 7758-01-2) was prepared in serum-free medium and added to the cells for 1 hour. Cells were then allowed to recover in serum-free medium containing either 0.1% DMSO or 10 μM TH5487 for 1, 4, or 6 hours. Cells were fixed with ice-cold acetone:methanol 1:1 (Fisher Scientific) for 20 min on ice and permeabilized with 0.5% Triton X-100 in phosphate-buffered saline (PBS) for 5 min. This was followed by RNAse treatment to degrade RNAs using the following buffer: 10 mM Tris-HCl (pH 7.5), 1 mM Ethylenediaminetetraacetic acid (EDTA), 0.4 mM NaCl, and 100 μg/mL RNAse (Zymo Research) for 1 hour at 37 °C. To make the 8-oxoG lesions accessible to the antibody, the DNA duplex was denatured by incubating the cells in freshly prepared 2.5 N HCl for 30 min at room temperature followed by a neutralization step using 0.1 M sodium borate Na_2_Bo_4_O_7_ (pH 8.8) for 10 min. Blocking with 4% bovine serum albumin (BSA; Sigma) in PBS was carried out for 1 h, followed by overnight incubation with anti-8-OHdG (Abcam, AB48508, N45.1) at 1:200. Incubation with the secondary antibody Alexa 647 (Thermo Fisher Scientific) was performed for 1 h at room temperature. 4′,6-diamidino-2-phenylindole (DAPI) was used to stain DNA. Imaging was performed with a Zeiss LSM 780 confocal microscope equipped with a UV-transmitting Plan-Apochromat 40×/1.30 Oil DIC M27 objective. The “tile scanning” and “positions” tools of ZEN software (ZEN, Zeiss, Germany) were used to acquire images from at least 9 positions per condition (unidirectional scanning, zoom 1, scan speed 10). Images were processed in ImageJ and Cell Profiler. For quantitative evaluation of 8-oxoG levels, the data of at least 500 cells from three independent experiments were averaged. The mean fluorescence intensity and the standard deviation were calculated and displayed using GraphPad Prism software.

### 2.4. In Situ Extraction

To study OGG1 retention under different experimental conditions, a pre-extraction treatment was performed to wash off soluble proteins loosely bound to the chromatin. U2OS cells stably expressing GFP-tagged OGG1 were treated with 0.1% Triton X-100 in PBS (Sigma) for 1 min prior to fixation with 4% paraformaldehyde (Santa Cruz). DAPI was used to stain the DNA for 10 min. Cells were then examined under a Zeiss LSM 780 confocal microscope equipped with a UV-transmitting Plan-Apochromat 63x/1.40 Oil DIC M27 objective. A 488 nm Ar laser was used to excite GFP. OGG1-GFP nuclear fluorescence signal intensities were recorded from three independent experiments. Images were processed in ImageJ and Cell Profiler. Data from at least 1300 cells for each treatment condition were assessed. The mean fluorescence intensity and the standard deviation were calculated and displayed using GraphPad Prism software.

### 2.5. Live Cell Microscopy, Laser Microirradiation and Fluorescence Recovery after Photobleaching

Live cell microscopy, microirradiation and fluorescence recovery after photobleaching (FRAP) experiments were performed as previously described by Xie et al. [15] and Visnes et al. [14]. In brief, U2OS cells stably expressing GFP-tagged wild-type OGG1 were seeded on Ibidi µ-dish (Ibidi #81166) 24 h prior to the indicated treatment. For laser microirradiation, cells were pre-sensitized with 10 μg/mL Hoechst 33342 (Thermo Fisher Scientific, Catalog No. 62249) for 10 min at 37 °C. To avoid background fluorescence from phenol red present in the DMEM culture medium, we exchanged the medium to live cell imaging medium (Thermo Fisher Scientific, Catalog No. 31053028) supplemented with penicillin–streptomycin antibiotics, 10% FBS and 25 mM HEPES containing either 0.1% DMSO or 10 μM TH5487 for 1 h. Cells were then transferred to a 37 °C pre-heated environmental chamber attached to a Zeiss LSM 780 confocal microscope equipped with a UV-transmitting Plan-Apochromat 40×/1.30 Oil DIC M27 objective. To induce DNA damage, a nuclear spot (dimensions: 10 × 10 pixels) was selected using the circular region tool of the ZEN software (ZEN, Zeiss, Germany) and irradiated using a 405 nm diode laser set to 100% (spot irradiation, 1 iteration, zoom 5, and pixel dwell time of 12.61 µs). For quantitative evaluation of the recruitment kinetics, the fluorescence intensity at the irradiated spot was corrected for background and for total nuclear loss of fluorescence over the time course and normalized to the pre-irradiation value. 

To induce oxidative stress for FRAP experiments, cells were challenged for 1 h with 40 mM potassium bromate (Sigma-Aldrich CAS 7758-01-2) dissolved in live cell imaging medium supplemented with penicillin–streptomycin antibiotics, 10% FBS and 25 mM HEPES (Gibco) as a pH buffer. This was followed by adding 0.1% DMSO or 10 μM TH5487 to the medium for another hour. The FRAP assay was performed according to the protocol described by Visnes et al. [14]. The data of at least 35 nuclei from three independent experiments were averaged. Images were processed in ImageJ. The mean curve and the standard error of the mean were calculated and displayed using GraphPad Prism software.

### 2.6. Quantitative Microscopy

For quantitative microscopy, cells were seeded in 96-well plates (10,000 cells/well; Corning 4680). Cells were treated with either 40 mM potassium bromate or 50 μM menadione and increasing concentrations of TH5487 or equal amounts of DMSO in serum-free DMEM. After 1 h of incubation, cells were pre-extracted with 0.1% Tritonx-100 in PBS for 1 min, fixed in 4% formaldehyde for 20 min, permeabilized with 0.5% Triton X-100 for 10 min, and probed with an anti-γH2AX antibody (Millipore, 05-636). Images were taken with an Image Xpress Micro (Molecular Devices) microscope using a 20× lens. Fluorescence intensities per cell nucleus were determined using a pipeline generated in Cell Profiler software and plotted using GraphPad Prism. 

### 2.7. Statistical Analysis

Data from two to three independent experiments were subjected to a two-tailed Student’s *t*-test to determine the statistical significance and are presented as means ± standard error of the mean (SEM) or standard deviation (SD) as indicated.

## 3. Results

### 3.1. TH5487 Treatment Results in Accumulation of Genomic 8-OxoG Lesions

**Figure 1:**
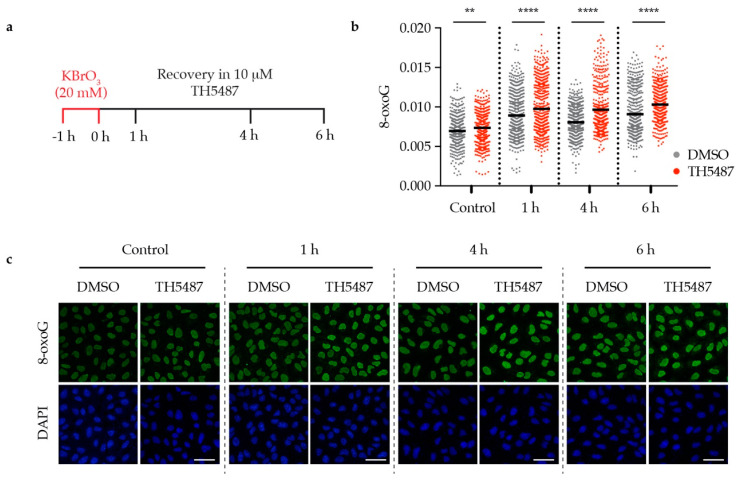
OGG1 inhibition with TH5487 results in accumulation of genomic 8-oxoG base lesions. (**a**) Scheme of TH5487 treatment to study the repair kinetics of genomic 8-oxoG. (**b**) Fluorescence signal intensity of 8-oxoG at the indicated time points. U2OS cells were pre-challenged with 20 mM KBrO_3_ for 1 h, then allowed to recover in a medium containing either 0.1% DMSO or 10 μM TH5487 for the indicated time points. Scatterplot shows data of at least 500 cells for each treatment condition with the mean from three independent experiments. Statistical significance was determined using an unpaired two-sided *t*-test (** *P* < 0.01, **** *P* < 0.0001). (**c**) Representative confocal images of U2OS cells showing accumulation of genomic 8-oxoG lesions. Scale bar: 50 μm.

We have previously described TH5487 as a selective small molecule inhibitor of OGG1 [14]. To further study the repair kinetics of 8-oxoG after inhibiting OGG1, we used the oxidizing agent potassium bromate (KBrO_3_), which has been reported to induce DNA oxidative damage, resulting in 8-oxoG lesions [16,17]. U2OS (osteosarcoma) cancer cells were pre-treated with 20 mM KBrO_3_ for 1 hour then released into fresh medium containing either 10 μM TH5487 or 0.1% DMSO. The levels of genomic 8-oxoG were found to be significantly higher in TH5487-treated cells at all time points, indicating that TH5487 prevents OGG1 from repairing its main substrate, 8-oxoG (Figure 1a–c). 

### 3.2. TH5487 Treatment Impairs OGG1 Binding to Damaged Chromatin

**Figure 2:**
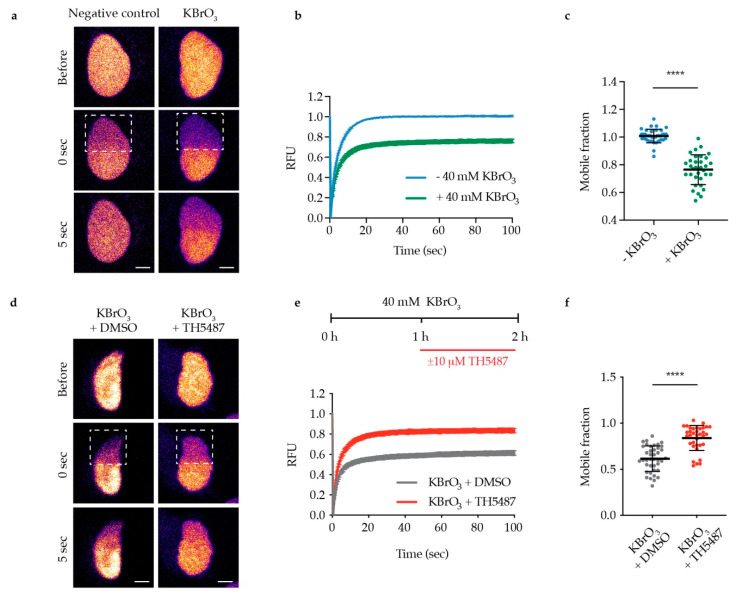
TH5487 alters OGG1–chromatin dynamics. (**a**) Representative false color images of nontreated and KBrO_3_-treated U2OS cells expressing OGG1-GFP are shown. Dashed outlines indicate bleached areas. Scale bar: 5 μm. (**b**) Quantification of fluorescence recovery after photobleaching (FRAP). U2OS cells expressing OGG1-GFP were treated or not with 40 mM KBrO_3_ for 1 h, a nuclear region was bleached, and recovery of fluorescence after photobleaching was recorded. RFU (relative fluorescence units). (**c**) Quantification of OGG1’s mobile fraction. Exposure to 40 mM KBrO_3_ for 1 h reduced the nuclear mobility of OGG1-GFP. (**d**) Representative false color images of DMSO-treated and TH5487-treated U2OS cells expressing OGG1-GFP are shown. Dashed outlines indicate bleached areas. Scale bar: 5 μm. (**e**) Quantification of FRAP experiments where U2OS cells expressing OGG1-GFP were treated with 40 mM KBrO_3_ for 1 h, followed by another hour of additional exposure to 10 μM TH5487 or 0.1% DMSO. (**f**) Quantification of OGG1’s mobile fraction. Treatment with TH5487 resulted in higher nuclear mobility of OGG1-GFP. Data are means ± SEM (b,e) or means ± SD (c,f) of three independent experiments. At least 35 cells from three independent experiments were quantified. Statistical significance was determined using unpaired two-sided *t*-tests (**** *P* < 0.0001).

**Figure 3:**
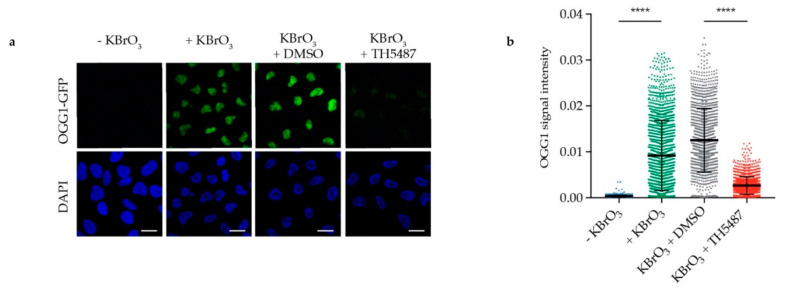
TH5487 treatment affects OGG1 retention in cells exposed to oxidative stress. (**a**) OGG1–chromatin binding. Representative confocal images of U2OS cells after in situ extraction. Scale bar: 20 μm. (**b**) Fluorescence signal intensity of retained OGG1-GFP. U2OS cells were exposed to the indicated treatment conditions, then in situ extraction with 0.1% Triton for 1 min was performed prior to fixation with 4% paraformaldehyde. Scatterplot shows OGG1-GFP nuclear fluorescence signal intensity of at least 1300 cells for each treatment condition and mean fluorescence intensity ± SD of three independent experiments. Statistical significance was determined using unpaired two-sided *t*-test (**** *P* < 0.0001).

The observed reduction in OGG1′s repair activity might arise from OGG1 being trapped on DNA. Alternatively, it might be due to OGG1 being unable to bind 8-oxoG because of its active site being occupied by TH5487. To test these hypotheses, we examined OGG1–chromatin binding using fluorescence recovery after photobleaching (FRAP) in U2OS cells stably expressing GFP-tagged OGG1. We observed strong OGG1 binding to chromatin after KBrO_3_ treatment (Figure 2a–c), indicating that such treatment results in oxidative DNA damage, to which OGG1 binds. Interestingly, OGG1 becomes more mobile in cells co-treated with KBrO_3_ and TH5487 (Figure 2d–f), suggesting that TH5487 prevents OGG1 from binding to damaged DNA. Potassium bromate has been shown to induce OGG1 retention on chromatin after in situ extraction [18]. Consistent with the FRAP results, more OGG1-GFP was found to be retained in U2OS cells treated with KBrO_3_ following in situ extraction, though this was reversed after concomitant treatment with KBrO_3_ and TH5487 (Figure 3). Taken together, these results show that TH5487 treatment impairs OGG1 binding to damaged chromatin. 

### 3.3. OGG1 Recruitment to Laser-Induced DNA Damage Sites is Reduced by TH5487 

**Figure 4:**
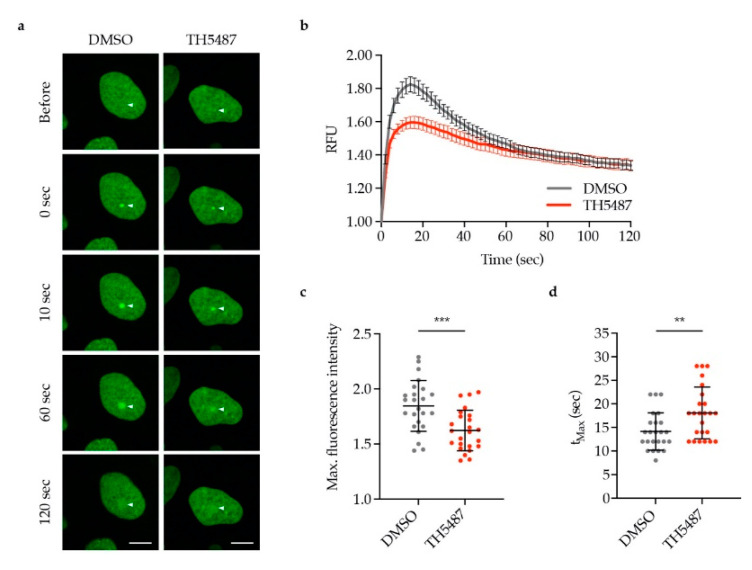
TH5487 treatment reduces OGG1 recruitment to laser-induced DNA damage sites. (**a**) Representative images of DMSO- and TH5487-treated U2OS cells expressing OGG1-GFP. Arrows indicate the point of laser irradiation. Scale bar: 10 μm. (**b**) OGG1 recruitment kinetics. U2OS cells expressing OGG1-GFP were pre-sensitized with Hoechst and treated with either 0.1% DMSO or 10 μM TH5487 for 1 h, then DNA damage was induced with a 405 nm laser. RFU (relative fluorescence units). (**c**) Quantification of the maximum fluorescence intensity upon OGG1-GFP recruitment. (**d**) Quantification of the time needed to reach maximum fluorescence of OGG1-GFP. The scatterplots show the data of at least 24 cells for each treatment condition. Data are means ± SEM (b) or means ± SD (c,d) of two independent experiments. Statistical significance was determined using unpaired two-sided *t*-tests (** *P* < 0.01, *** *P* < 0.001).

To investigate whether the reduced OGG1–chromatin binding observed in TH5487-treated cells was caused by lower recruitment or faster dissociation of OGG1, we monitored OGG1-GFP recruitment to laser-induced DNA damage sites. OGG1-GFP recruitment kinetics were found to be impaired in TH5487-treated cells exemplified by recruitment of less OGG1-GFP (Figure 4a–c) and the longer time needed for the fluorescence intensity to reach its maximum level (Figure 4d). 

### 3.4. TH5487 Impairs OGG1 Incision and Generation of DNA Breaks

**Figure 5:**
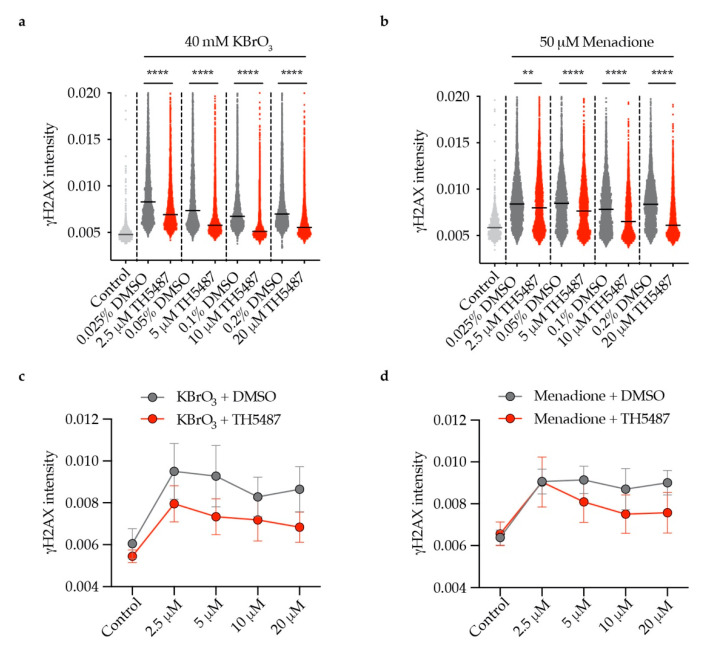
Concentration-dependent reduction of OGG1 incisions by TH5487. (**a**,**b**) Reduced nuclear γH2AX intensity in U2OS cells treated with (**a**) KBrO_3_ or (**b**) menadione and increasing concentrations of TH5487 for 1 h. (**c**,**d**) Mean values of (a) and (b). Scatterplots show data of at least 1000 (control) or 3300 (all other conditions) cells. Data are presented as median (a,b) and mean (c,d) ± SEM of two independent experiments with two technical repeats each. Statistical significance was determined using unpaired two-sided *t*-tests (** *P* < 0.01, **** *P* < 0.0001).

Excision of 8-oxoG lesions by OGG1 can result in the generation of DNA single-strand breaks (SSBs) [19]. Near-simultaneous attempted base excision repair (BER) of clustered oxidative lesions on opposing strands may generate double-strand breaks (DSBs) [20,21,22]. We sought to study whether TH5487 treatment impairs OGG1′s incision activity in cells, affecting DNA break generation. U2OS cells expressing OGG1-GFP were co-treated with oxidizing agents, KBrO_3_ or menadione and increasing concentrations of TH5487 or equivalent amount of DMSO. Both oxidizing agents have been reported to induce oxidative stress, generating 8-oxoG lesions [16,17,23]. Quantitative microcopy revealed that γH2AX formation is significantly reduced in a dose-dependent manner by TH5487 (Figure 5). This indicates that TH5487 not only prevents the binding of OGG1 to damaged DNA, but also inhibits its catalytic function in cells, leading to fewer incisions and thus fewer breaks.

## 4. Discussion

Although cancer cells are inherently stressed by high levels of ROS and DNA damage, DNA pathways repairing oxidative DNA damage have not been extensively targeted by inhibitors as a therapeutic approach [13]. The success of PARP inhibitors in pre-clinical and clinical studies supports the potential significance of new inhibitors for BER [24,25,26]. DNA glycosylases in particular may serve as promising anticancer targets, as mice lacking these enzymes are generally viable and fertile [27]. The high load of ROS in cancer cells and the relative abundance of guanines in GC-rich promoters and telomeres [28,29,30] have prompted us and others to develop the first small-molecule OGG1 inhibitors [14,31]. 

We have previously shown that TH5487 inhibits OGG1′s glycosylase activity in in vitro biochemical assays. In addition, T cell leukemia Jurkat A3 cells treated with KBrO_3_ and released into fresh medium containing TH5487 showed an increase in genomic 8-oxoG levels as detected by liquid chromatography–tandem mass spectrometry (LC-MS/MS) [14]. Consistently, modified comet assays demonstrated that genomic OGG1 substrates accumulate in Jurkat A3 cells treated with TH5487, as such treatment caused an increase in OGG1-induced tail length [32]. Notably, TH5487 treatment suppresses the growth of a large panel of cancer cell lines encompassing several human solid tumors including osteosarcoma, colon, glioblastoma, kidney and lung cancer but is well tolerated by non-transformed cells [32]. Here, we sought to further characterize the effect of TH5487 on cancer cells using a solid tumor cell model, U2OS. We observed that OGG1 inhibition results in 8-oxoG accumulation in DNA (Figure 1). This confirms that TH548 targets OGG1′s glycosylase activity in U2OS cells and that the previously described elevation in 8-oxoG levels upon TH5487 treatment [14] is not restricted to Jurkat A3 T lymphocytes.

Live cell imaging and FRAP assays in U2OS cells show that upon TH5487 treatment, OGG1 is more mobile (Figure 2d–f), indicating that OGG1 binding to damaged chromatin is impaired. This affirms the observed phenotype in A3 cells, where TH5487 was found to alter OGG1–chromatin dynamics [14]. The increased mobility of OGG1 excludes any entrapment of OGG1 on damaged DNA after OGG1 inhibition. This is in agreement with an almost complete lack of detectable OGG1 retention in pre-extracted cells treated with both KBrO_3_ and TH5487 (Figure 3). Importantly, the observed impaired OGG1–chromatin binding, together with the previously reported in vitro electrophoretic mobility shift assay (EMSA) results, support our conclusion that TH5487 hampers OGG1 binding to damaged DNA. Besides, it complements the reported crystal structure of mouse OGG1 in complex with one analogue of TH5487 confirming targeting of OGG1′s active site [14]. The recently resolved crystal structure of human OGG1 in complex with TH5487 further confirms that TH5487 targets the active site of human OGG1. OGG1 adopts a closed conformation upon binding to TH5487, which blocks OGG1′s access to its substrate DNA lesions [32]. This explains the increased mobility of OGG1 upon TH5487 treatment (Figure 2d–f).

TH5487 interferes with OGG1 binding to damaged DNA and affects its recruitment kinetics to DNA damage sites as well (Figure 4). Hence, this strongly suggests that the impaired OGG1–chromatin binding observed in inhibitor-treated cells (Figure 2 and Figure 3) is not a result of faster OGG1 dissociation from DNA. This provides an additional explanation as to why more 8-oxoG lesions are detected in oxidatively stressed cells upon treatment with TH5487 (Figure 1). 

During BER of clustered oxidative DNA lesions existing on opposing strands, double-strand breaks can be introduced [20,21,22]. Our observation that the level of γH2AX is reduced in TH5487-treated cells (Figure 5) illustrates that the compound inhibits OGG1′s incision activity, verifying the reported effect of TH5487 on OGG1′s enzymatic activity in in vitro biochemical assays [14]. This inhibition is translated into accumulation of genomic 8-oxoG lesions as shown in Figure 1. A definitive quantitative method for 8-oxoG is lacking. Hence, the absolute level that cancer or normal cells can withstand is not yet clear. Further investigation is needed to avoid on-target toxicity in normal tissues. 

As TH5487 selectively induces proliferation arrest in cancer cell lines but not in non-transformed cells [32], OGG1 serves as a potential target for cancer treatment. More research should be directed towards optimizing the compound’s pharmacokinetic profile to determine its efficacy in in vivo cancer models.

## 5. Conclusions

This study validates TH5487 as a potent OGG1 inhibitor that prevents the repair of 8-oxoG lesions and alters both OGG1–chromatin dynamics and OGG1′s recruitment kinetics to DNA damage sites. We demonstrate that TH5487 can be used to pharmacologically inhibit the glycosylase activity of OGG1, enabling future studies of OGG1′s role in different disease models.
